# PSA and Beyond: The Past, Present, and Future of Investigative Biomarkers for Prostate Cancer

**DOI:** 10.1100/tsw.2010.182

**Published:** 2010-10-01

**Authors:** Jeffrey Tosoian, Stacy Loeb

**Affiliations:** James Buchanan Brady Urological Institute, Johns Hopkins Medical Institutions, Baltimore, MD, USA

**Keywords:** prostate cancer, biomarkers, screening, diagnosis, prostatic acid phosphatase, prostate-specific antigen (PSA), PSA density, free PSA, PSA kinetics, PSA isoforms, PCA3, GSTP-1, AMACR, single nucleotide polymorphism (SNP)

## Abstract

The discovery of prostate-specific antigen (PSA) as a biomarker represented a major discovery in the early diagnosis and monitoring of prostate cancer. However, the use of PSA is limited by the lack of specificity and an inability to differentiate indolent from life-threatening disease reliably at the time of diagnosis. A multitude of studies have aimed to improve the performance of PSA as well as identify additional biomarkers. The purpose of this study is to review available data on prostate cancer biomarkers for prostate cancer screening and prognostication, including prostatic acid phosphatase, PSA, PSA derivatives (PSA density, free PSA, pro PSA, and PSA kinetics), PCA3, GSTP1, AMACR, and other newly emerging molecular and genetic markers.

## HUMAN PROSTATIC ACID PHOSPHATASE

In 1935, Kutscher and Wolbergs discovered an acid phosphatase that was present in high amounts in the human prostate[1]. A subsequent study by Gutman et al. revealed a relationship between this prostatic acid phosphatase (PAP) and prostate cancer, showing that serum PAP levels were elevated in men with metastatic disease[2]. This group additionally demonstrated that acid phosphatase activity was increased at sites of prostate cancer metastases in bone[3]. As a result of these early studies, PAP was acknowledged as the first known biomarker for prostate cancer[4].

The significance of discovering this marker was described by Huggins and Hodges, who used PAP activity to indicate the success or failure of hormonal therapy[5]. Later studies supported the use of PAP in combination with clinical findings to predict outcomes[6,7], but attempts to use the enzyme in diagnosis remained largely unsuccessful[8]. As such, the diagnosis of prostate cancer remained a purely clinical endeavor and serum levels of PAP were considered only after diagnosis.

With the introduction of prostate-specific antigen (PSA) in the 1980s, PAP was rendered largely obsolete. In addition to improved specificity, PSA was shown to have greater utility in screening and prognostication[9]. More recently, however, there has been renewed interest in serum PAP as a possible prognostic marker[10]. In one recent study of men with higher-risk prostate cancer, pretreatment PAP outperformed PSA and Gleason score in predicting cancer-specific survival after treatment[11]. With further investigation in a contemporary setting, PAP may ultimately prove useful in specific clinical contexts.

## PSA

Secreted by prostatic epithelial cells, PSA is a 33-kDa glycoprotein that functions in the liquefaction of seminal fluid. PSA was originally described by Hara et al. in forensic studies as a marker for human semen[12]. It was later found to be present in normal benign hypertrophic and malignant prostatic tissue[13]. Studies from the Roswell Park Memorial Institute demonstrated that PSA was detectable in human serum and elevated in men with prostate cancer[14,15]. By the mid-1980s, evidence emerged that PSA was superior to PAP in monitoring prostate cancer after treatment[16], and PSA testing was approved by the Food and Drug Administration for use in this setting. Multiple studies demonstrated that PSA was a sensitive marker for detecting residual disease after treatment and tumor recurrence during follow-up[17,18]. An undetectable level of PSA after radical prostatectomy is used today to indicate the absence of recurrence[19] and serial PSA measurements are also used in the contemporary setting to define recurrence after definitive radiation therapy[20].

Beginning in the early 1990s, multiple studies suggested that serum PSA may be useful as a tool in prostate cancer screening[21,22,23]. In a multicenter clinical trial of 6,630 men, Catalona et al. reported that PSA, when used in conjunction with digital rectal examination (DRE), enhanced early prostate cancer detection[24]. These and other findings led to FDA approval of PSA for prostate cancer screening, using a threshold of 4.0 ng/ml.

The introduction of large-scale PSA-based screening was associated with dramatic increases in prostate cancer incidence throughout the 1990s[25]. Moreover, a greater proportion of men were diagnosed with early-stage disease and far fewer presented with distant metastases[26]. This “stage migration” is well documented in the Surveillance, Epidemiology, and End Results (SEER) database, wherein the proportion of men with metastases at presentation decreased from 16 to 4% with screening[27]. Indeed, a large analysis by Jemal et al. suggested that widespread PSA testing was associated with decreased incidence of late-stage disease and prostate cancer–specific mortality[28].

Using 4.0 ng/ml as the threshold for biopsy, Catalona et al. reported that a significant proportion of cancers had spread to the prostate capsule by the time of detection[29]. Furthermore, longitudinal analysis revealed that of men with PSA ≥2.5 ng/ml, approximately one-half demonstrated a PSA increase to 4.0 ng/ml within 4 years[30]. As such, the authors decreased the threshold for biopsy to 2.5 ng/ml in their screening study[31]. Later, they demonstrated a direct relationship between PSA levels at diagnosis and the likelihood of organ-confined disease[32]. In men with preoperative PSA levels of 2.6–4.0, 4.1–7.0, 7.1–10.0, and >10 ng/ml, the rates of organ-confined disease were 81, 74, 72, and 60%, respectively, and 10-year recurrence-free survival estimates differed significantly across preoperative PSA strata (log-rank test, $p = 0.0001$). This study provided evidence that cancers detected at PSA levels of 2.6–4.0 ng/ml have higher rates of organ-confined disease and improved 10-year recurrence-free survival than those detected at higher PSA levels.

Additional trials provided support for the use of a lower PSA threshold for biopsy. In 2004, Thompson and colleagues discovered cancer in 17% of men with low PSA (1.1–2.0 ng/ml) and a normal DRE, indicating that even the most stringent biopsy criteria would miss a significant proportion of cancers[33]. Alarmingly, this trial demonstrated high-grade disease in 14.9% of the cancers detected at a PSA ≤4.0 ng/ml.

A problem with the use of a lower PSA threshold for biopsy is that PSA may be elevated as a result of various nonmalignant conditions, e.g., benign prostatic hyperplasia and prostatitis, or after iatrogenic manipulation of the prostate, such as transrectal ultrasound[34]. These confounders may lead to unnecessary biopsies and this problem would be compounded by lowering PSA thresholds. Even using the conventional threshold, 75% of men with PSA 4.0–10.0 ng/ml who undergo biopsy do not have cancer, introducing a significant source of unnecessary cost, as well as potential anxiety and morbidity[35]. At the same time, studies have indicated that PSA screening leads to the detection of some cancers that would have otherwise remained undetected during life[36]. This is concerning, as unnecessary treatment of indolent cancers may be associated with significant morbidity, especially in older men[37].

There has been a great deal of investigation aimed at improving the accuracy of PSA-based screening. Adjunctive measurements considering the rate of PSA changes with time, the ratios of free and protein-bound PSA, and the relationship of PSA to prostate size have improved performance characteristics in some settings[38]. Several nomograms combining PSA with other clinical variables have also been developed to improve prognostic value beyond that of individual tests[39]. At the same time, there has been increased interest in active surveillance programs for low-risk patients, presenting an option to reduce the extent of unnecessary overtreatment[40,41].

Amid the many questions surrounding PSA, a most fundamental question remains: Is PSA-based screening beneficial? Despite the evidence presented above and elsewhere, this question came under increased scrutiny when two large trials demonstrated contradictory findings. Preliminary results of a U.S. trial comparing annual screening to usual care found no significant difference in cause-specific mortality[42], although there were numerous methodological limitations to this study. Conversely, in a large European trial, Schroder et al. demonstrated a 20% reduction in cause-specific mortality in the screened arm vs. the control arm[43]. Considering these findings in combination with the available literature, PSA-based screening has undoubtedly contributed to the significant decline in prostate cancer-mortality rates observed in the U.S. and abroad since the late 1990s[44,45]. Nonetheless, there is continued debate as to whether the benefits of prostate cancer screening outweigh its risks.

## PSA DERIVATIVES

### Free PSA

Serum PSA circulates in either an unbound “free” form or bound to one of several proteins, most frequently alpha-1-antichymotrypsin (ACT)[46,47]. Levels of free PSA (fPSA) can be detected and compared to total PSA, yielding the proportion of free PSA (%fPSA). Studies have shown that men with the highest proportions of complexed PSA are more likely to have prostate cancer[48] and that %fPSA is lower in men with prostate cancer as compared to benign prostatic hyperplasia (BPH)[49]. Thus, %fPSA showed promise in distinguishing malignant from benign prostate disease.

In addition to investigating the function of fPSA, follow-up studies helped to establish its performance characteristics. In 1995, Prestigiacamo and Stamey demonstrated a median %fPSA of 8.9 in men diagnosed with prostate cancer and 16.5 in men with BPH[50]. In the same year, Luderer et al. published a comparative study including men with prostate cancer, benign prostate disease, as well as healthy asymptomatic controls[51]. Among all subjects, both total PSA and %fPSA differentiated cancer from benign conditions. Furthermore, %fPSA outperformed total PSA in men in the “diagnostic gray zone” (PSA = 4.0–10.0 ng/ml). Using a threshold fPSA value of 25%, another study of men with intermediate PSA levels yielded 95% sensitivity and 20% specificity for prostate cancer diagnosis[52]. When restricted to men with total PSA of 3.0–7.0 ng/ml, the use of a 20% fPSA threshold improved specificity to 38%.

One potential benefit of using %fPSA is reducing the proportion of unnecessary biopsies. In 1998, a prospective multicenter trial showed that %fPSA reduced unnecessary biopsies by 20% when using a threshold fPSA of 25%[53]. This trial also showed that, in general, cancers detected at fPSA >25% were smaller and lower grade. Other trials similarly demonstrated a reduction in the unnecessary biopsy rate, most commonly using %fPSA thresholds ranging from 20 to 27%[31]. In light of these data, fPSA was approved by the FDA for use in the screening and diagnosis of prostate cancer at PSA levels between 4.0 and 10.0 ng/ml.

Numerous studies have explored methods of optimizing diagnostic accuracy of fPSA. One such study demonstrated a higher %fPSA in larger prostates, suggesting clinical adjustment of threshold values based on prostate volume[54]. Subsequent trials identified other challenges to the use of fPSA, such as conditions at the time of sample obtainment, *in vitro* instability, and interassay variability[50,55,56]. These factors may help to explain inconsistencies in the performance of fPSA[57]. Despite known limitations, a comprehensive meta-analysis demonstrated that %fPSA significantly outperformed total PSA in predicting biopsy outcomes in men with intermediate PSA[58]. Considering the available data, fPSA appears to be a useful tool for diagnosis, particularly in men with intermediate levels of serum PSA.

## PSA Density

To enhance the diagnostic accuracy of PSA, Benson et al. described the concept of PSA density (PSAD) as the ratio of PSA concentration to prostate volume[59]. This initial study found significant differences in mean PSAD between men with prostate cancer vs. BPH (0.581, 0.044; $p < 0.002$), identifying the potential utility of PSAD in differentiating prostate cancer from benign disease. Subsequent studies evaluated PSAD in additional clinical settings, with many demonstrating a modest improvement in diagnostic accuracy when PSAD was considered in addition to PSA[60,61].

Because the transition zone is primarily involved in BPH, subsequent studies investigated the value of adjusting PSA to transition zone volume rather than total prostate volume. A study by Kalish et al. found that PSA density of the transition zone (PSADTZ) was more predictive of positive biopsy than conventional PSAD in patients with intermediate PSA levels (4.0–10.0 ng/ml)[62]. Kikuchi et al. similarly demonstrated that PSADTZ was superior to PSAD in distinguishing cancer from BPH[63]. Despite these positive findings, other studies concluded that PSAD had value only in limited patient subgroups, and still others failed to identify a single practical use for PSAD or PSADTZ that improved predictive performance in screening[64,65].

Other studies have demonstrated the utility of PSAD in predicting clinicopathological features of disease, such as Gleason score and total cancer volume at radical prostatectomy[66]. Similarly, on multivariate analysis, Radwan et al. found that PSAD independently predicted positive surgical margins, extracapsular extension, seminal vesicle invasion, and biochemical failure after prostatectomy[67]. These findings supported earlier data suggesting that PSAD could predict tumor pathology in men with nonpalpable prostate cancer[68]. More recently, Tseng et al. demonstrated that PSA density may aid in predicting the risk of progression in men on active surveillance[69]. In the contemporary setting, PSAD may be most effective when used with other clinical factors to stratify risk and weigh treatment options. A drawback of PSAD is the need for transrectal ultrasound, which often is not performed as a separate procedure prior to prostate biopsy, as well as limitations in the accuracy and interexaminer variability in prostate size measurement.

## PSA Isoforms fPSA exists in multiple molecular isoforms, including a BPH-related isoform termed BPSA[70,71], inactive PSA (iPSA), and proPSA, which has been associated with prostate cancer[74,75]. ProPSA was originally described with a 7-amino-acid leader sequence and was subsequently found to circulate in other forms with leader sequences of 5, 4, and 2 amino acids[72,73].

In 2003, a study of men with total PSA levels of 2.0–10.0 ng/ml demonstrated that the percentage of proPSA (%pPSA), calculated as proPSA divided by fPSA, was more specific for detecting prostate cancer when compared to complexed PSA or fPSA alone[76]. In men with PSA of 2.5–4.0 ng/ml, Sokoll et al. similarly showed %pPSA outperformed %fPSA in detecting prostate cancer and reducing unnecessary biopsies[77]. Subsequently, the focus shifted to the [-2]proPSA (p2PSA) isoform, which was recently tested in a large European population[78]. Other studies have shown that in men with PSA ≤10.0 ng/ml, the proportion of p2PSA (%p2PSA) significantly outperformed %fPSA in diagnosing prostate cancer[79,80].

In addition to improving diagnostic accuracy, one subsequent trial revealed that %pPSA selected for higher-grade disease and extracapsular extension[81]. More recent investigations of %pPSA have had similarly encouraging results. For example, a study of men with low-risk prostate cancer in active surveillance demonstrated that levels of serum and tissue proPSA at presentation were significantly associated with progression to unfavorable biopsy findings during follow-up[82]. Thus, proPSA may be useful in the surveillance setting to predict men who will eventually require curative intervention.

More recently, proPSA has been incorporated into a proprietary equation known as the Beckman Coulter Prostate Health Index (phi), which is calculated as: (p2PSA pg/ml / fPSA ng/ml) × (PSA ng/ml)$^{½}$. In one study, phi offered the greatest discrimination for prostate cancer on receiver operating characteristic analysis. Additional investigation on the role of proPSA and phi are currently underway.

## PSA Kinetics

The calculation of changes in PSA over time was first described by Carter et al. using data from the Baltimore Longitudinal Study of Aging[83]. Using comprehensive longitudinal data, Carter and colleagues revealed greater increases in PSA with time (PSA velocity, PSAV) in men who were eventually diagnosed with prostate cancer when compared to men who were never diagnosed with prostate cancer. Importantly, these differences in PSAV were observed several years prior to clinical diagnosis, conferring the potential for very early identification of disease. Meanwhile, serum PSA levels at this time did not differentiate between the groups. Using a cutoff of 0.75 ng/ml/year, PSAV distinguished men with prostate cancer from BPH and healthy controls with a specificity of 90 and 100%, respectively. Further analysis revealed that longitudinal PSA measurements followed an exponential growth pattern in men who were later diagnosed with cancer[84]. This shift in growth curves occurred at an average of 7–9 years prior to clinical diagnosis, also suggesting a role of PSAV in early detection.

Another method of quantifying changes in PSA over time, PSA doubling-time (PSADT), is defined as the time it takes for serum PSA to double. Initial studies of PSADT demonstrated its utility in characterizing the biological significance of disease recurrence after treatment. In 1999, Pound et al. demonstrated that a postoperative PSADT <10 months predicted worse metastasis-free survival[19]. Follow-up studies demonstrated that lower PSADT was associated with increased risk of cancer-specific mortality in men with PSA recurrence after surgery[85] and radiation therapy[86].

Many recent studies have examined the role of PSA kinetics in prognostication. One such study revealed that PSAV predicts the likelihood of clinically significant cancer in men with PSA levels ≤4.0 ng/ml[87]. Another study in men with normal PSA levels demonstrated an association between PSAV and long-term prognosis. In this analysis, Carter et al. showed that PSAV measured 10–15 years prior to prostate cancer diagnosis was associated with rates of cancer-specific mortality 25 years later[88]. Two significant studies by D'Amico et al. showed that pretreatment PSAV >2 ng/ml/year was associated with increased prostate cancer–specific mortality after either radiation therapy or radical prostatectomy[89,90].

Despite its success in predicting prognosis, the utility of PSA kinetics in screening has become increasingly controversial[91]. In 2006, Thompson et al. reported that PSA kinetics did not reliably predict a diagnosis of prostate cancer in men with PSA ≤4.0 ng/ml[92]. Similarly, a study of 1,699 men with PSA levels <10.0 ng/ml demonstrated that PSADT and PSAV had little to no value in predicting biopsy results[93]. Furthermore, data from the European Randomized Study of Screening for Prostate Cancer (ERSPC) demonstrated that PSAV was not an independent predictor of positive biopsy[94]. Thus, some critics have concluded that despite the prognostic value, PSA kinetics have little value in predicting biopsy results[95,96,97].

Ultimately, the inconsistencies of PSA kinetics may be partially explained by differences in methodology. For example, predictive performance can vary greatly depending on data points used in calculating kinetic values. One study recommended using at least three measurements obtained over 2 years for accurate calculation[98]. Kinetic values based on measurements obtained in the short term (e.g., <6 months apart) may be less useful due to individual fluctuations in serum PSA levels[99]. Furthermore, differences in PSA assay standardization may also affect calculations in the real-world setting[100]. In an effort to standardize their use and optimize predictive ability, there has recently been greater attention focused on data collected for kinetic studies[101]. Nonetheless, the concept of PSA kinetics has improved the prognostic value of longitudinal data beyond the capabilities of PSA measurements alone.

## PCA3

In 1999, Bussemakers et al. described DD3 (also known as PCA3) as a potential biomarker for prostate cancer[102]. Additional studies from this group characterized DD3 as a noncoding, prostate-specific mRNA that is highly overexpressed in prostate cancer[103]. Initial studies demonstrated high sensitivity and specificity for prostate cancer, as DD3 was overexpressed in 94.6% of cancerous tissue samples and not in controls. Follow-up studies corroborated DD3 as a highly specific tissue marker, revealing insignificant DD3 expression in benign tissue and in nonprostatic tumors. Importantly, mRNA was expressed at 34-fold increased levels in malignant vs. nonmalignant tissue.

In a subsequent study, Hessels et al. used RT-PCR to demonstrate a 66-fold increase of DD3 expression in prostate tumors[104]. RT-PCR was then used to detect DD3 in urine, revealing a sensitivity of 67% and a negative predictive value of 90%. In a more recent cohort study, Marks et al. found that DD3 was superior to PSA in predicting prostate cancer in men with elevated PSA and a previous negative biopsy[105]. At the same time, there is evidence that the PCA3 score is associated with clinical and pathological features. Urine PCA3 scores demonstrated a significant association with extracapsular extension, tumor volume, and Gleason score[106,107]. A relationship between PCA3 and Gleason score was similarly demonstrated in a European population[108].

With a better understanding of its clinical strengths and limitations, PCA3 may serve as a valuable biomarker moving forward. Observed associations with pathologic tumor features suggested a potential role in selecting or monitoring men with low-risk cancers. As such, our institution recently assessed the relationship of urinary PCA3 and repeat biopsy results in an active surveillance population[109]. Analysis revealed higher PCA3 scores in men with high-risk cancer on surveillance biopsy, particularly in those upgraded based on Gleason score, but differences were not statistically significant. Furthermore, a sensitive and specific PCA3 threshold for biopsy could not be identified due to considerable overlap of PCA3 scores between groups. Although its role in surveillance remains unclear, PCA3 has shown great promise in the general population as a diagnostic and prognostic marker for prostate cancer.

## GSTP1

Enzymes of the glutathione S-transferase (GST) family have many functions in cellular metabolism, notably including the detoxification of potentially harmful substrates[110]. In 1994, Lee et al. demonstrated hypermethylation of promoter sequences at the *GSTP1* gene in human prostatic cancer tissue specimens[111]. Additionally, the authors observed a substantial decrease in *GSTP1* expression associated with prostatic cancers, in contrast to abundant *GSTP1* expression in normal prostatic epithelium. These and other studies led to the hypothesis that *GSTP1*-encoded enzymes serve as “caretakers” of prostatic cells, and that failure to express these enzymes due to somatic CpG hypermethylation plays a role in prostatic carcinogenesis[112]. Nonetheless, *GSTP1* promoter hypermethylation has been observed in >90% of prostatic cancers, making it the most common DNA alteration associated with prostate cancer[112,113,114].

Because tumor DNA can be detected in the bodily fluids of some prostate cancer patients[115,116], Goessl et al. utilized methylation-specific PCR to evaluate *GSTP1* promoter hypermethylation in various samples obtained from men with prostate cancer[117]. The authors observed *GSTP1* promoter hypermethylation at rates of 94% in tissue, 72% in serum, 50% in ejaculate, and 36% in urine. Remarkably, hypermethylation was found in 100% of serum specimens from patients with locally advanced or metastatic disease. By comparison, hypermethylation was absent in all tissue samples and bodily fluids obtained from men with biopsy-confirmed BPH.

DNA-based molecular tumor markers have potential for very high sensitivity and specificity, theoretically reaching 100%[118]. A review of other *GSTP1* methylation studies in urine revealed a consistently high specificity, ranging from 93 to 100%[117,119,120,121,122,123,124]. Nevertheless, detection rates in these studies were not sufficiently reliable, ranging from 19 to 76%. Prostate cancer detection rates based on plasma samples were similarly inadequate, ranging from 13 to 72%[117,121,122]. Furthermore, very few studies have identified *GSTP1* promoter hypermethylation in ejaculate, and detecting cancer biomarkers in ejaculate has traditionally been very difficult[125]. Ultimately, however, the identification of a molecular marker for prostate cancer represented a major scientific advance. With improved sensitivity, *GSTP1* promoter hypermethylation may prove to be a useful marker in prostate cancer detection.

## AMACR

Advances in molecular biology have allowed for the discovery of other novel biomarkers for prostate cancer. Using cDNA library subtraction and high-throughput microarray screening, Xu et al. demonstrated overexpression of alpha-methylacyl coenzyme A racemase (AMACR) in prostate cancer at both the mRNA and protein levels[126]. AMACR is an enzyme with a well-characterized role in the beta-oxidation of branched-chain fatty acids and bile acid intermediates[127]. A potential relationship between this enzyme and prostate cancer development is particularly intriguing because the main sources of branched-chain fatty acids in humans, including beef and dairy products, have been implicated as dietary risk factors for prostate cancer[128].

In 2001, Jiang et al. assessed AMACR as a molecular biomarker for prostate cancer[129]. The authors used a monoclonal antibody to stain 137 prostatic cancer tissue samples and 70 benign prostate tissue samples. They reported positive expression of AMACR in all 137 prostate cancer specimens, and analysis revealed a sensitivity of 100% and specificity of 88%. A study by Rubin et al. in 2002 similarly demonstrated overexpression of AMACR in prostate cancer[130]. In this study, AMACR expression in prostate biopsy specimens detected cancer with 97% sensitivity and 100% specificity. An additional study by Luo et al. demonstrated that AMACR and p63 antibodies could be used in combination to further enhance the accuracy of prostate cancer diagnosis[131]. The high sensitivity and specificity of AMACR staining holds great promise for making a diagnosis when conventional staining methods are inconclusive[132].

## Genetic Markers

The recent advent of genome-wide association studies has revolutionized the field of prostate cancer genetics. More than 30 single nucleotide polymorphisms (SNPs) have been identified across the human genome with demonstrated associations with prostate cancer susceptibility. For example, SNPs on multiple regions of chromosome 8q24 now have well-validated associations with prostate cancer risk[133]. In conjunction with two alleles on chromosome 17, these variants were shown to have a cumulative relationship with prostate cancer risk[134]. Specifically, carriers of five risk alleles with a positive family history of prostate cancer had a 9.5-fold increased risk of prostate cancer, as compared with noncarriers, in a landmark study by Zheng et al.[134]. Many other SNPs have since been identified with links to prostate cancer susceptibility, although evidence has been less convincing on the relationship between many of these alleles and disease aggressiveness[135,136]. Because genetic susceptibility testing is becoming commercially available, additional study is urgently needed to determine whether these new markers have a role in prostate cancer screening and risk stratification.

## CONCLUSION

The use of biomarkers for prostate cancer extends back nearly a century, but it was forever changed in the 1980s with the discovery of PSA. Serum PSA demonstrated effectiveness as a marker for recurrence after treatment and it continues to be used for this purpose today. Since the initiation of widespread PSA-based screening, most contemporary patients are diagnosed with early-stage prostate cancer that is curable with treatment. Despite this, the value of PSA in screening has come under scrutiny due to its lack of specificity and potential for the overdiagnosis of nonthreatening cancers. To improve performance characteristics, a number of PSA derivatives and isoforms have also been studied. These biomarkers have shown promising, but mixed, results.

More recently discovered biomarkers, such as DD3 (PCA3), GSTP1, and AMACR, have favorable performance characteristics in some clinical settings. In order to best utilize these markers, however, additional study is needed to define the appropriate context and optimal parameters for their use. Until the discovery of a biomarker with both high sensitivity and specificity, screening will likely continue to involve a joint consideration of multiple markers in combination with other clinical factors.

At the same time, emerging genetics discoveries may represent a new revolution in prostate cancer research. In the future, genomic testing may allow improved identification of men at risk for prostate cancer, or may help to guide the use of existing biomarkers and personalize screening protocols.

Despite numerous limitations, there have been great strides in prostate cancer biomarker research in recent years. Ongoing investigation into these and other new markers in the future will continue to improve our ability to stratify prostate cancer risk and prognosis.
